# Half-Metallic Behavior in Doped Sr_2_CrOsO_6_ Double Perovskite with High Transition Temperature

**DOI:** 10.1038/srep15010

**Published:** 2015-10-08

**Authors:** Kartik Samanta, Prabuddha Sanyal, Tanusri Saha-Dasgupta

**Affiliations:** 1 Department of Condensed Matter Physics and Material Sciences, S.N. Bose National Centre for Basic Sciences, JD Block, Sector-III, Salt Lake City, Kolkata 700 098, India; 2 Department of Physics, Indian Institute of Technology, Roorkee 247667, India

## Abstract

Half-metallic magnets with metallic behavior in one spin channel and insulating in the other, have attracted considerable attention due to their potential application possibility. The spin-dependent nature of the carrier scattering due to half-metallic nature of these materials, allows for the resistance to be strongly influenced by the low magnetic field. However, the operating temperatures of such known materials are generally low, opening up the need for half-metallic magnets with high transition temperatures. The double perovskites having general formula A_2_BB′O_6_ with alternating ordered arrangement of two transition metal sites, B and B′ offer an attractive possibility in this respect. Here, we consider the case of Sr_2_CrOsO_6_, which is a ferrimagnetic insulator with transition temperature (T_*c*_) of 725 K, highest ever known in the oxide family, and show that moderate amount of La and Na doping at Sr site can drive the compound half-metallic with high T_*c*_.

Double perovskites (A_2_BB′O_6_) consisting of alkaline-earth or rare-earth metal ion at A site, and two different transition metal ions at B and B′ sites, are of significant interest due to the diverse properties exhibited by them, which include multiferroicity^1^, magnetodielectric behavior^2^,^3^, large magneto-optic responses^4^ etc. Rock-salt ordering of B and B′ ions can be achieved for large size and valence difference between B and B′ cations^5^. The observation of high magneto-resistance in half-metallic double perovskite Sr_2_FeMoO_6_ with fairly high transition temperature^6^ of 410 K indicates the promise in double perovskite materials as candidates for high temperature half metallic magnets^7^. The wide-spread use of magnetic materials, however, is limited by the extent of demagnetization at ambient conditions. One therefore needs to be sufficiently below T_*c*_ for the full moment to develop. This underlines the need to have materials with T_*c*_ as much higher than room temperature as possible, so that room temperature applications can be made. It is therefore of crucial importance to search and understand the occurrence of magnetic materials with high T_*c*_. In this search of such materials, the Cr-based double perovskite Sr_2_CrOsO_6_ is worth investigating which shows the magnetic transition temperature of ≈725 K^8^. This compound though is an insulator^8^ with gap in the calculated density of states in both spin channels^9^. A natural question that arises, starting from such a material with spectacularly high T_*c*_, whether it is possible to drive the half-metallic state, yet maintaining high magnetic transition temperature. In the present study, we explore this possibility by La and Na doping at Sr site. Previous study^10^,^11^,^12^ of La doping in Sr_2_FeMoO_6_ showed that novel states can be obtained through doping carriers in the pristine compound, motivating the present study. We note that substitution of Sr by La amounts to electron doping while substitution of Sr by Na amounts to hole doping. We consider six different doped compounds, Sr_1.875_La_0.125_CrOsO_6_, Sr_1.75_La_0.25_CrOsO_6_, Sr_1.625_La_0.375_CrOsO_6_, and Sr_1.875_Na_0.125_CrOsO_6_, Sr_1.75_Na_0.25_CrOsO_6_, Sr_1.625_Na_0.375_CrOsO_6_. Our first-principles density functional theory (DFT) based calculations together with exact diagonalization of Cr-Os model Hamiltonian constructed in a first-principles derived Wannier function basis show that through the route of chemical doping, half-metallic, ferrimagnetic state is achievable with reasonably large net magnetic moment of ≈0.5–1.0 *μ*_*B*_ and magnetic transition temperature nearly as high as the parent compound. Our prediction of half-metallicity in doped Sr_2_CrOsO_6_ with high T_*c*_ should be a viable scheme to design magneto-resistive devices operative at room temperature.

## Results

### DFT electronic and magnetic structures

**Figure 1:**
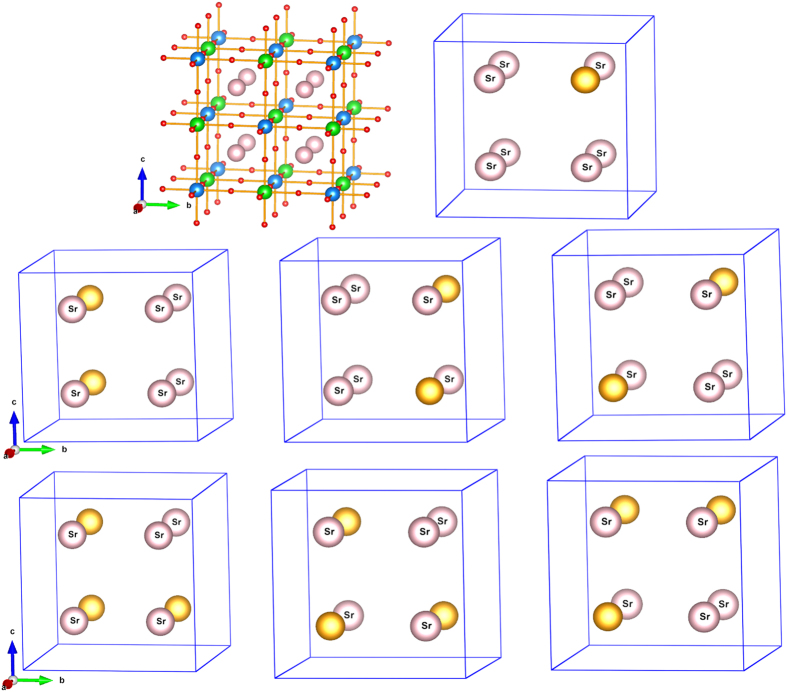
Top row, left panel: The cubic structure of Sr_2_CrOsO_6_ in Fm-3m space group. The large shaded-brown, medium green, medium blue and small red balls represent Sr, Cr, Os and O atoms respectively. Top row, right panel: The *A* sublattice with one out of eight Sr atoms substituted by Na/La. The substituted atom is shown as yellow ball. Middle row: The *A* sublattice with two out of eight Sr atoms substituted by Na/La, in various inequivalent positions. Bottom row: The *A* sublattice with three out of eight Sr atoms substituted by Na/La, in various inequivalent positions.

**Figure 2:**
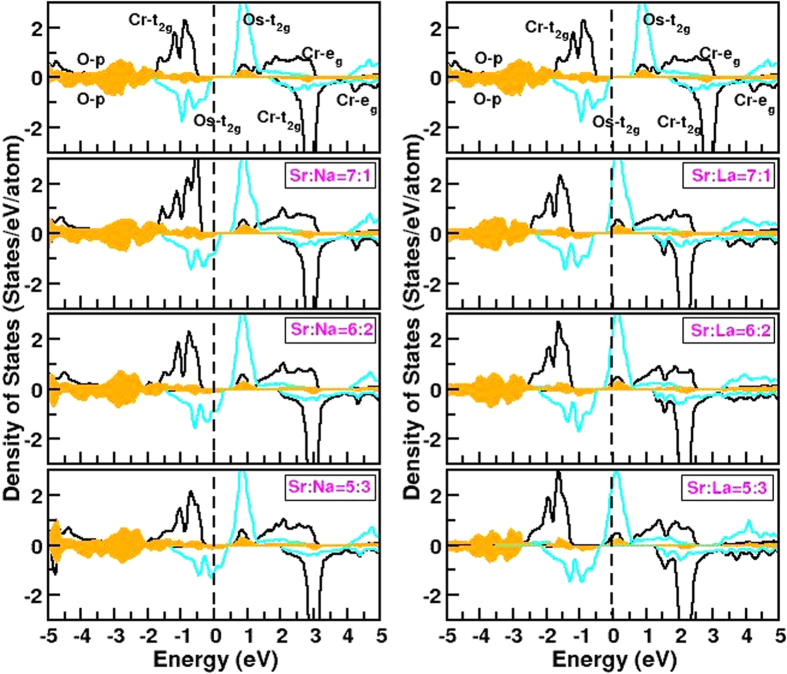
Left panel: GGA + *U* + SOC density of states of the parent compound, and the Na doped compounds. From top to bottom, the various panels refer to parent compound, Sr_1.875_Na_0.125_CrOsO_6_, Sr_1.75_Na_0.25_CrOsO_6_, Sr_1.625_Na_0.375_CrOsO_6_ compounds respectively. The zero of the energy marks the position of E_*F*_. The black, cyan, yellow shaded area represent the states projected to Cr *d*, Os *d* and O *p* states, respectively. Right panel: Same as left panel, but shown for parent and La doped compounds. From top to bottom, the various panels refer to parent compound, Sr_1.875_La_0.125_CrOsO_6_, Sr_1.75_La_0.25_CrOsO_6_, Sr_1.625_La_0.375_CrOsO_6_ compounds respectively. The dashed, vertical lines in each panel mark the positions of Fermi level.

In order to study the doping effect of La and Na at the Sr site of Sr_2_CrOsO_6_, we considered the parent compound in its cubic Fm-3m space group^8^, and constructed a supercell 4 times larger than that of the Fm-3m primitive unit cell. This created eight Sr sites, out of which one, two and three Sr atoms were replaced by La/Na. For the case of two and three atom substitutions, different inequivalent configurations were considered, as shown in Fig. 1. After substitution of Sr by La/Na, the structure was optimized completely both in terms of volume and atomic positions. The calculated spin-polarized electronic structure is presented in Fig. 2. The electronic structure of the two and three atom substituted cases are averaged over different inequivalent configurations.

As is evidenced from Fig. 2, the states close to Fermi level (E_*F*_) are dominated by Cr and Os *d* states hybridized with O *p* states, while the O *p* dominated states separated from Cr and Os *d* dominated states occupy the energy range far below E_*F*_. Sr *s* and *d* dominated states (not shown in figure) remain far above E_*F*_. The *d* states of Cr and the Os ions are exchange split as well as crystal field split. For the parent compound, in the majority spin channel, Cr *t*_2*g*_ states are filled while the Cr *e*_*g*_ states remain empty with the empty Os *t*_2*g*_ states lying in between Cr *t*_2*g*_ and Cr *e*_*g*_ states. In the minority spin channel, the Os *t*_2*g*_ states remain completely filled, separated from Cr *t*_2*g*_, Cr *e*_*g*_ and Os *e*_*g*_, thus giving rise to an insulating solution with gap in both spin channels. We notice finite hybridization of Os t_2*g*_ states with Cr *t*_2*g*_ states. Upon increasing Na doping, the filled Os *t*_2*g*_ states in the minority spin channel get progressively depleted, thus moving away from the gapped situation for the parent compound. This leads to a half-metallic solution with finite density of states at E_*F*_ in the minority spin channel and a gap in the majority spin channel. In case of La doping, the added electrons via La doping progressively fill up the majority spin Os *t*_2*g*_ states which happened to be empty in the parent compound. This again gives rise to half-metallic solution with finite density of states at E_*F*_ in the majority spin channel and a gap in the minority spin channel. Thus both hole doping and electron doping via Na and La doping, respectively, leads to half-metallic solutions. From the calculated density of states at T = 0 K, the value of the gap in the insulating spin channel turns out to be about 0.6–0.7 eV for Na doped compounds, and about 1.5–1.6 eV for La doped compounds, giving rise to the expectation that half-metallic character will be retained even at significantly high temperature below T_*c*_. We note however, the nature of the carriers is rather different in two cases, in case of hole (Na) doping the carriers belong to Os-Cr hybridization derived rather broad *t*_2*g*_ bands having a band width of about 2 eV, while for electron (La) doping they belong to sharply peaked Os *t*_2*g*_ majority bands having a width of about 0.5 eV or so. Thus the mass of the carriers in electron and hole doping is expected to be different, as will be reflected in transport measurement.

**Figure 3:**
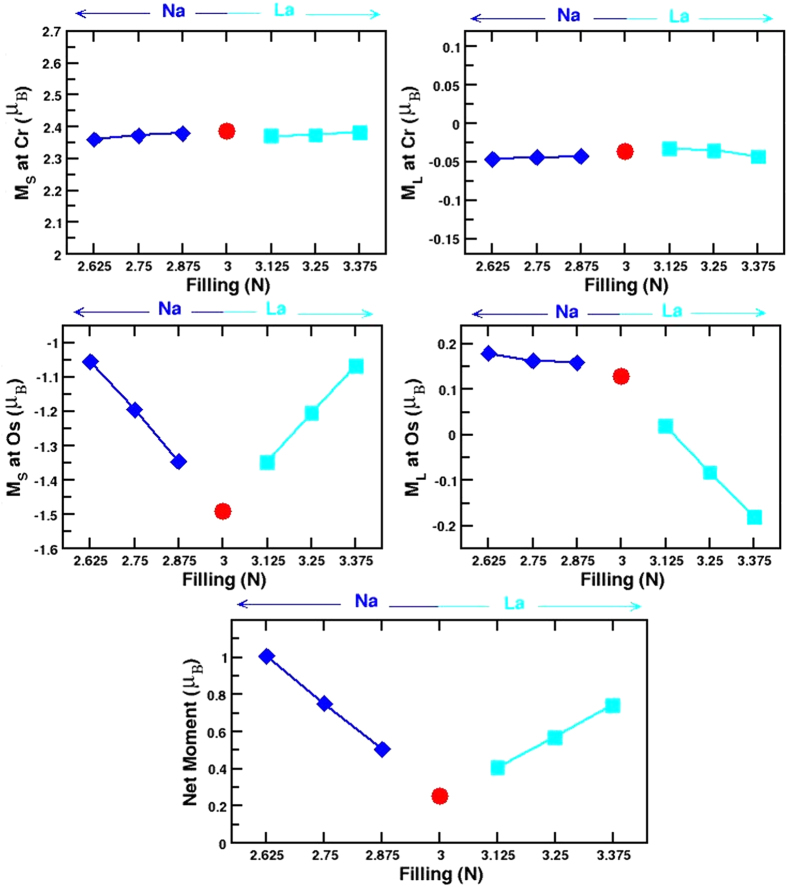
Top panels: Calculated M_*S*_ and M_*L*_ at Cr site for the parent and six different doped compounds as a function of the the nominal valence electron count of Os ion. The circle denotes the parent compound, while the diamond and squares represent Na doped and La doped compounds. Middle panels: Same as top panel, but shown for that at Os site. Bottom panel: The net magnetic moment per formula unit plotted as a function of the nominal valence electron count of Os.

We find the half-metallicity achieved by Na/La doping is robust upon the inclusion of spin-orbit coupling. This is unlike the case of Sr_2_CrReO_6_ where the half-metallicity was reported to be destroyed by inclusion of spin-orbit coupling^13^. The calculated spin (M_*s*_) and orbital moments (M_*L*_) plotted as a function of the nominal electron count at Os site, are shown in Fig. 3. We find that both spin and orbital moments at Cr site remain more or less unchanged upon doping Na or La, as the doped carriers go to Os rather than to Cr. The tiny orbital moment at Cr site points in the opposite direction to that of spin moment, in agreement with half-filled nature of Cr *t*_2*g*_ states. The spin and orbital moments at Os site show interesting trend upon doping as Os site hosts the doped carriers. The absolute value of the spin moment at Os site decreases systematically from that of the parent compound by both Na and La doping due to decrease in number of unpaired Os *t*_2*g*_ electrons. We find the decrease to be symmetric with respect to hole and electron doping. The orbital moment at Os in the parent compound is expected to be quenched due to half-filled Os *t*_2*g*_ states, but finite orbital moment is obtained which is argued to be driven by covalency with O and empty *e*_*g*_ states^14^. La as well as Na doping, shifts the valence of Os away from half-filled 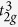 configuration, which in turn increases the value of orbital moment. The orbital moment points in opposite direction to the spin moment for the parent and Na doped compounds, for which the nominal filling of Os is 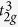 or less. La doping leads to nominal filling of Os larger than 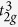 which makes the orbital moment at Os site pointing in the same direction as the spin moment. The effect of decreased moment at Os site for both Na and La doping, makes the net moment grow due to antiferromagnetic nature of coupling between Os and Cr. In absence of spin-orbit coupling, the parent compound ideally should have been an antiferromagnet or, more suitably termed as a compensated ferrimagnet with zero net moment. The presence of non negligible orbital moment at Os site makes the parent compound an uncompensated ferrimagnet with a net moment^8^,^9^. The introduction of doped carriers and increase of doping level, weakens the compensation between Cr and Os moments more and more, resulting into a sharp increase in the net moment, which reaches a value of about 1 *μ*_*B*_ and 0.7 *μ*_*B*_ for 18.75% Na and La doping, respectively, the largest doping level considered in the present study.

In connection to role of spin-orbit, we notice that M_*L*_/M_*s*_ at Os site is much less than 1. As discussed, the nominal valence of Os in the studied compounds is 5+ or close to 5+ (ranging from 4.875+ to 5.375+) state, with low-spin *d* occupancy of 3 or close to 3 (ranging from 3.125 to 2.625) thus making orbital contribution of the moment to be small. Additionally, the presence of strongly magnetized ion as Cr, and the three dimensional magnetic connectivity between the 3*d* ion (Cr) and the 5*d* ion (Os) influences the size of the spin moment on the Os site, thereby further reducing the M_*L*_/M_*s*_. This was discussed in the context of La_2_CoIrO_6_^15^, though the case of La_2_CoIrO_6_ for which Ir is in nominal 4+ valence state and thus in *d*^5^ configuration is different from the compounds under discussion.

### Exact Diagonalization Study of DFT derived Model Hamiltonian

In order to estimate the magnetic T_*c*_, it is essential to estimate the magnetic exchanges, which can be obtained from total energy calculations of different spin configurations. This approach in the context of Sr_2_CrOsO_6_ is faced with complexity. The magnetism in Sr_2_CrOsO_6_, as explained in ref. 9, arises due to interplay of the hybridization driven mechanism and super-exchange mechanism. Energetically, the *t*_2*g*_ levels of Os fall within the exchange split *t*_2*g*_ levels of Cr. Switching on the hybridization between Cr and Os, allows the states of same symmetry and spin to interact. This leads to a renormalized spin splitting at Os site, the direction of which is opposite to that of Cr. This in turn ensures the parallel alignment of Cr spins, driven by the hybridization mechanism^16^. It was further shown in ref. 9 that Os has intrinsic moment in addition to the induced moment by hybridization with Cr. This intrinsic moment leads to superexchange interaction involving Os intrinsic spins. The presence of the intrinsic moment at Os site makes the moment at Os site frustrated in an antiferromagnetic configuration of Cr spins. The hybridization driven mechanism, on the other hand, disfavors stabilization of magnetic configurations with Cr spins aligned in parallel to the spins at Os sites. This in turn leads to strong frustration effect in some of spin configurations, requiring consideration of different possible non-collinear, canted spin configurations, as shown in ref. 17 obtained from Monte Carlo simulation of a classical spin Hamiltonian. It is difficult to handle such cases within full blown DFT scheme. We thus consider model Hamiltonian approach in the following, which allows one to calculate the physical properties in a much more manageable way.

The model Hamiltonian is defined in N-th order muffin tin orbital Wannier basis and solved with exact diagonalization for the ferromagnetic (FM) and paramagnetic (PM) configurations of Cr spins. The PM phase is simulated as disordered local moment calculations, where the calculations are carried out for several (≈50) disordered configurations of Cr spin and are averaged to get the energy corresponding to paramagnetic phase. We note here such disordered calculation would have been extremely difficult to carry out within DFT owing to the computational cost involved using large supercells, and also averaging them over several configurations. The difference of the two energies gives the estimate of transition temperature.

**Figure 4:**
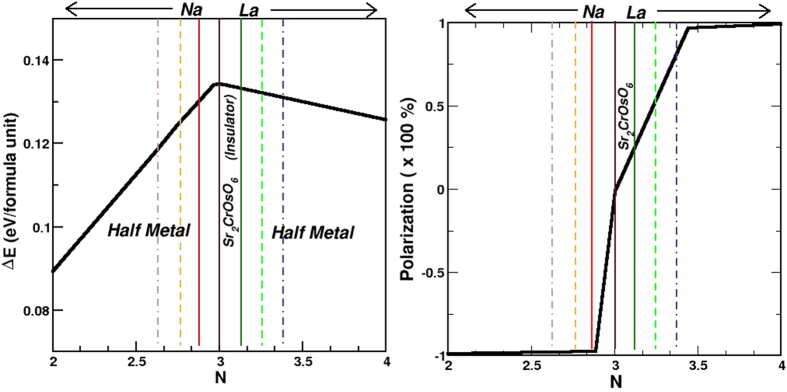
Left panel: The energy difference between PM and FM arrangement of Cr spins, plotted as a function of the valence electron count of Os ion, shown as black solid line. The vertical lines mark the filling corresponding to the parent (solid, magenta) and various Na (red-solid, orange-dashed and brown-dot-dashed for Sr_1.875_Na_0.125_CrOsO_6_, Sr_1.75_Na_0.25_CrOsO_6_, Sr_1.625_Na_0.375_CrOsO_6_, respectively) and La doped compounds (green-solid, green-dashed and indigo-dot-dashed for Sr_1.875_La_0.125_CrOsO_6_, Sr_1.75_La_0.25_CrOsO_6_, Sr_1.625_La_0.375_CrOsO_6_, respectively) studied in present work. Both the Na-doped and La-doped compounds are half-metallic, while the parent compound with electron count of three, is insulating. Right panel: The spin polarization plotted as a function of the electron occupancy of Os ion. The vertical lines mark the parent and various Na and La doped compounds as in left panel.

The model Hamiltonian, describing the interplay of the hybridization and super-exchange mechanism is given by,


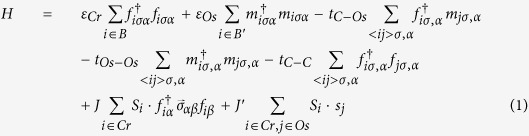


where the *f*’s and *m*’s refer to the Cr *t*_2*g*_ and Os *t*_2*g*_ degrees of freedoms. *t*_*C*−*Os*_, *t*_*Os*−*Os*_, *t*_*C*−*C*_ represent the nearest neighbor Cr-Os, second nearest neighbor Os-Os and Cr-Cr hoppings respectively. *σ* is the spin index and *α* is the orbital index that spans the *t*_2*g*_ manifold. The difference between the ionic levels, Δ = *ε*_*Cr*_ − *ε*_*Os*_, defines the on-site energy difference between Cr *t*_2*g*_ and Os *t*_2*g*_ levels. *s*_*j*_ is the intrinsic moment at the Os site. The first six terms of the Hamiltonian describe the hybridization mechanism, or equivalently a double-exchange like mechanism, consisting of a large core spin at the Cr site (*S*_*i*_) and the coupling between the core spin and the itinerant electron delocalized over the Cr-Os network. The last term represents the superexchange mechanism in terms of coupling between Cr spin and the intrinsic moment at Os site. The parameters of the model Hamiltonian are extracted out of DFT calculations through NMTO downfolding technique of constructing the real space Hamiltonian in the basis of effective Cr *t*_2*g*_ and Os *t*_2*g*_ degrees of freedom, by integrating out all other degrees of freedom other than Cr and Os *t*_2*g*_’s. The DFT estimates of Δ, *t*_*C*−*Os*_, *t*_*Os*−*Os*_ and *t*_*Cr*−*Cr*_ are found to be 0.26 eV, −0.35 eV, −0.12 eV and −0.08 eV respectively, with little variation between the parent and doped compounds. *J* and *J*′ parameters are obtained from the spin splitting at Cr site and the extra splitting at Os site than that expected from solely hybridization mechanism^9^. This Hamiltonian is solved by exact diagonalization^18^ on a lattice of 8 × 8 × 8. See the supplementary information for further technical details on the solution of the model Hamiltonian. The energy difference between FM and PM configuration of Cr spins for the parent as well as doped compounds plotted as a function of Os *t*_2*g*_ filling (N) is shown in the left panel of Fig. 4. We find that the energy difference to be maximum for the undoped compound, and decreasing for both electron doping and hole doping. However the decrease is asymmetric, the decrease being more rapid for the hole doping, compared to electron doping. This shows the complex nature of magnetic interactions in this series of compounds, and also the speciality^19^ of the electron filling N = 3. Decrease of delocalized carriers by hole doping weakens the hybridization driven mechanism, thus reducing the PM-FM energy difference. Extrapolating the data for N = 0 gives the PM-FM energy difference to be zero, as for N = 0 there is no delocalized carrier to support the magnetism. Upon electron doping, the competing antiferromagnetic (AFM) ordering of B-site spins becomes effective^20^ as discussed for La doped Sr_2_FeMoO_6_^10^, which reduces the PM-FM energy difference. The large Os-Os hopping, on the other hand prevents the AFM ordering of Cr spins winning over the FM. This stabilizes the FM ordering of Cr spins in La doped Sr_2_CrOsO_6_, as opposed to the case of La doped Sr_2_FeMoO_6_^10^,^12^ for which La doping is found to stabilize a metallic state with AFM ordering of Fe spins. T_*c*_ values can be estimated by mapping the PM and FM energy difference to the mean field formula. While the mean field approximation is expected to overestimate the T_*c*_ significantly, the ratio’s of T_*c*_’s are expected to reproduced rather well, as demonstrated in ref. 9. We find that doped compounds maintain the high T_*c*_ properties, with 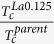 = 0.99, 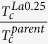 = 0.98, 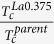 = 0.97, 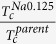 = 0.96, 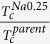 = 0.92, and 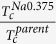 = 0.87.

Another important issue is the degree of spin polarization. The most popular definition is given by *P* = (*N*_↑_ − *N*_↓_)/(*N*_↑_ + *N*_↓_) where *N*_↑(↓)_ is the value of the density of states in two spin channels. Using this definition the spin polarization is 100% for any half-metal. This definition, however, ignores the mass of the carriers, which should be reflected in transport measurement. A more suitable definition in this respect is given by^21^, 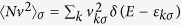 where *ε*_*kσ*_ is the band energy and 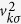 is the corresponding velocity at wavevector *k*. We calculated the spin polarization from model Hamiltonian calculations. For this purpose, first the quasiparticle dispersion is obtained from the model Hamiltonian using a variational ansatz for the spin state, then the quantity X = 〈*Nv*^2^〉 is calculated for both spin channels. The polarization, *P* is obtained as *P* = (*X*_↑_ − *X*_↓_)/(*X*_↑_ + *X*_↓_). In this context, it is worth mentioning that spin-orbit effect can change the degree of spin-polarization. See for example, discussion in ref. 22. However, in the discussed compounds the configuration of Os is *d*^3^ or close to *d*^3^. This leads to a rather small effect of spin-orbit, with M_*L*_/M_*S*_ values much less than 1 (see Fig. 3). The spin-orbit coupling was thus not considered in the calculation of *P*. The right panel of Fig. 4 shows the plot of spin polarization plotted as a function of the electron count at Os site. The signs of the spin polarizations are opposite for La and Na doping, as carriers originate from the two different spin channels in two cases. Importantly, we find that the degree of spin polarization is rather different between La doped and Na doped compounds. This arises due to the fact, that Na doping produces carriers in the broad Os *t*_2*g*_ – Cr *t*_2*g*_ hybridized bands in the minority spin channels, and thus producing light carriers with faster speed. La doping, on the other hand produces carriers in the relatively narrow Os *t*_2*g*_ bands in the majority spin channel, thus producing heavier carriers with slower speed. Na doping in this respect may be advantageous over La doping, though the transition temperatures are expected to be higher for La doped compounds.

## Discussion

To summarize, our DFT calculations together with exact diagonalization of the low energy model Hamiltonian consisting of Cr and Os *t*_2*g*_ degrees of freedom led us to conclude that moderate La or Na doping of about 10 to 20% at the Sr site of Sr_2_CrOsO_6_ results into half-metallic ferrimagnetic behavior with high transition temperature which is estimated to be 87 to 99% of the T_*c*_ of the parent compound, reported to be ≈725 K^8^. Our calculation shows the effect of doping is not symmetric with respect to La, and Na doping which dopes electrons and holes, respectively. This asymmetry arises due to the positioning of Os *t*_2*g*_ energy levels with respect to that of Cr *t*_2*g*_ states, which makes Os *t*_2*g*_ states closer to Cr *t*_2*g*_ minority states and further from Cr *t*_2*g*_ majority states. Due to this asymmetry, we find that the magnetic transition temperatures to be in general higher for La doped compounds compared to Na doped compounds, while the degree of spin polarization is higher in Na doped compounds compared to La doped compounds. The optimal choice could be to consider the Na doping of about 10% which has both high T_*c*_ and high degree of spin polarization. Finally, La doping reduces the valence difference between Cr and Os making the ordering of Cr and Os presumably harder compared to the parent compound, which may lead to increased antisite disorder. Though La doping in Sr_2_FeMoO_6_ has been experimentally achieved^12^ for high La doping of 75% making the situation rather promising. The situation is expected to be opposite for Na doping which increases the valence difference between Cr and Os. We hope that our computational study will encourage experimental investigations in this direction. The enigmatic re-entrant metal insulator transition as a function of electron filling as one increases the filling from 2 (Na-doped) to 4 (La-doped) also deserves attention.

Finally, we notice that the present situation of doped Sr_2_CrOsO_6_ is very similar to what has been recently proposed in literature in the context of a 2-band model Hamiltonian^23^. In this study, the special case of correlated band insulator was considered with antiferromagnetic order at half-filling in which the up and down spins are at inequivalent sites, leading to different spectral gaps for the two spin components. Upon doping, according to results of ref. 23, one obtains a half-metallic ferrimagnet. In absence of spin-orbit interaction, Sr_2_CrOsO_6_ is a compensated ferrimagnet with two distinctly different gaps in the majority and minority spin channels. Thus the case of Sr_2_CrOsO_6_ is similar to the model study of ref. 23, the role of two inequivalent sites being played by Cr and Os sites, if the effect of spin-orbit is kept aside. The additional effect of spin-orbit at Os site in case of Sr_2_CrOsO_6_ makes the undoped Sr_2_CrOsO_6_ an uncompensated ferrimagnet with a net magnetic moment, rather than a compensated ferrimagnet with zero magnetic moment. In this respect, Sr_2_CrOsO_6_ differs from the model study in ref. 23 but the essential physics upon doping is found to be similar to that described in ref. 23, with spin-orbit playing minor role. Our study thus forms realization of the model study proposed in ref. 23 which can be validated experimentally.

## Methods

Our first-principles calculations are based on Linearized Augmented Plane Wave (LAPW) method as implemented in the Wien2k code^24^ with no shape approximation to the potential and charge density. For the number of plane waves in LAPW calculations, the criterion used is muffin-tin radius multiplied by K_*max*_ (for the plane wave) yielding a value of 7.0. Results have been cross-checked in terms of calculations in plane wave basis as implemented in the Vienna Ab-initio Simulation Package (VASP)^25^,^26^ with projector-augmented wave (PAW) potential^27^. The kinetic energy cut-off for expansion of wavefunctions used is 450 eV, and reciprocal space integrations are carried out with a k-space mesh of 8 × 8 × 8. The exchange-correlation functional is chosen to be that given by generalized gradient approximation (GGA)^28^. Both Cr and Os being transition metal ions correlation effect necessarily arise and GGA alone may not sufficient to take into account of this. The correlation effect beyond GGA at the transition metal sites in our calculations is taken into account through supplemented on-site Hubbard *U* correction via GGA + *U* formulation^29^. Cr being 3rd transition metal and Os being 5d transition metal with wider bands compared to Cr, it is expected correlation effect will be stronger in Cr compared to Os. We have thus chosen *U* values of 4.8 eV and 1.8 eV at 3*d* Cr and 5*d* Os sites, respectively. The HundÂ´s coupling parameters *J*_*H*_ is chosen to be 0.8 eV, giving rise to *U*_*eff*_ = *U* – *J*_*H*_ = 4 eV and 1 eV on Cr and Os sites, respectively. The trend of our results does not depend on chosen U value. Spin-orbit coupling (SOC) important due to presence of Os ions is included in the calculations in scalar relativistic form as a perturbation to the original Hamiltonian. The inclusion of *U* was found to be necessary for opening up a clear band gap in the parent compound in presence of SOC. For extraction of a few-band, tight-binding Hamiltonian out of full DFT calculation, to be used as input to exact diagonalization study, we carry out muffin-tin orbital (MTO) based NMTO-downfolding calculations^30^. The reliability of the calculations in different basis sets has been checked.

The results of exact diagonalization calculations are carried out on a lattice of dimension 8 × 8 × 8. Calculations have been carried out as well for lattices of different sizes and the trend is found to be the same as that presented.

## Additional Information

**How to cite this article**: Samanta, K. *et al.* Half-Metallic Behavior in Doped Sr_2_CrOsO_6_ Double Perovskite with High Transition Temperature. *Sci. Rep.* **5**, 15010; doi: 10.1038/srep15010 (2015).

## Supplementary Material

Supplementary Information
