# Supplementary material for: Half-Metallic Behavior in Doped Sr2CrOsO6 Double Perovskite with High Transition Temperature
Source: Sci Rep. 2015 Oct 8;5:15010. doi: 10.1038/srep15010 (PMC4597228; doi:10.1038/srep15010)
Supplement: Supplementary Information [file srep15010-s1.pdf]

Half-Metallic Behavior in Doped Sr_2CrOsO_6 Double Perovskite with High Transition Temperature

Kartik Samanta[1], Prabuddha Sanyal[2], and Tanusri Saha-Dasgupta[1]

1 *Department of Condensed Matter Physics and Material Sciences,*

S.N. Bose National Centre for Basic Sciences, JD Block,

Sector-III, Salt Lake City, Kolkata 700 098, India. and

2 *Department of Physics, Indian Institute of Technology, Roorkee 247667, India*

We start with the model Hamiltonian as given in the manuscript,

$$\begin{aligned} H &= \epsilon_{Cr} \sum_{i \in B} f^{\dagger}_{i\sigma\alpha} f_{i\sigma\alpha} + \epsilon_{Os} \sum_{i \in B'} m^{\dagger}_{i\sigma\alpha} m_{i\sigma\alpha} \\ &- t_{C-Os} \sum_{<ij>\sigma,\alpha} f^{\dagger}_{i\sigma,\alpha} m_{j\sigma,\alpha} - t_{Os-Os} \sum_{<ij>\sigma,\alpha} m^{\dagger}_{i\sigma,\alpha} m_{j\sigma,\alpha} \\ &- t_{C-C} \sum_{<ij>\sigma,\alpha} f^{\dagger}_{i\sigma,\alpha} f_{j\sigma,\alpha} + J \sum_{i \in Cr} \mathbf{S}_i \cdot f^{\dagger}_{i\alpha} \vec{\sigma}_{\alpha\beta} f_{i\beta} \\ &+ J' \sum_{i \in Cr, j \in Os} \mathbf{S}_i \cdot \mathbf{s}_j \end{aligned} \tag{1}$$

As mentioned in the manuscript, the onsite energies ϵ's and hopping parameters t's are obtained from NMTO-downfolding calculation. The few band Hamiltonian in the basis of Cr and Os effective t_{2g} orbitals is obtained by starting from a non spin-polarized full DFT calculation and integrating out the O and Sr, as well as Cr and Os e_g degrees of freedom. The real space representation of this Hamiltonian provides the estimates of ϵ's and t's, which are the spin-independent components of the model Hamiltonian, given in Eqn.(1). The tight-binding bands described by ϵ's and t's in comparison to the non-spinpolarized DFT bands are shown in Fig. S1. In absence of any spin-polarization this leads to partially filled Cr t_{2g} and Os t_{2g} bands of d^3 occupancies with metallic character.

The spin-polarization in the model Hamiltonian arises from last two terms in Eqn(1) governed by parameters J and J'. These two exchange parameters have been determined following the procedure given in Ref 9 of the main text, which involves the spin splitting at Cr site and the extra splitting at Os site than that expected from solely hybridization mechanism, as obtained from a magnetic downfolding calculation, as opposed to nonmagnetic downfolding calculation, which provides information of ϵ's and t'. Given the fact that $J \gg t_{C-Os}$, this Hamiltonian can be cast into form appropriate for $J \to \infty$. This has been done by performing a rotation to the local $\mathbf{S}_i$ axis at each Cr site, and retaining only the electron state oriented antiparallel to $\mathbf{S}_i$ at that site. This gives the Hamiltonian, with 'spinless' Cr conduction electrons and Os electrons having both spin degrees of freedom. This is the lowest energy Hamiltonian. There is no longer any 'large' coupling in the model, and the number of degrees of freedom has been reduced to three per Cr site and six per Os, compared to original problem with six degrees of freedom at both the sites. Since the spin **S** is large and can be considered classical, one can consider different spin configurations (ferro, antiferro, canted and disordered) and diagonalize the system in real space, to obtain variational estimates of the ground state, and its stability.

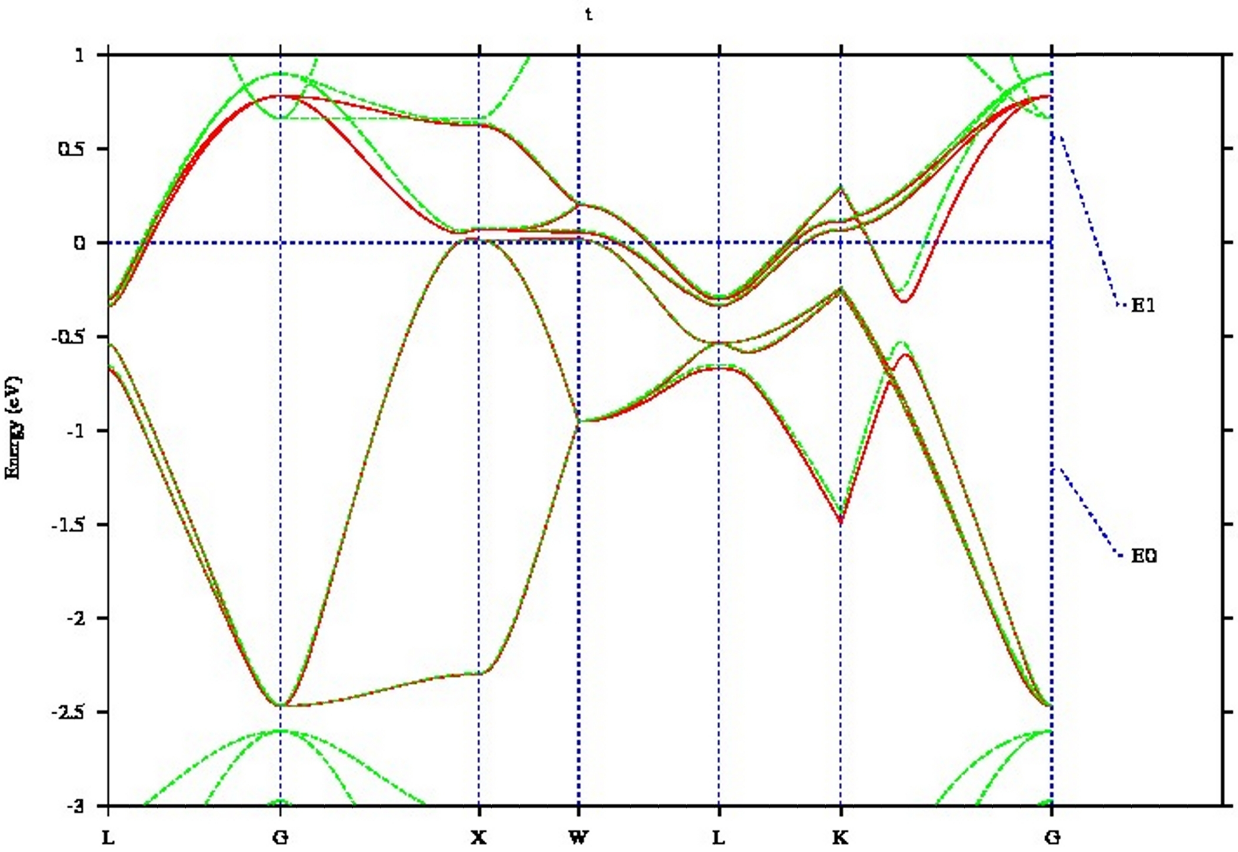

FIG. 1: The downfolded, tight-binding band structure of Sr_2CrOsO_6 in effective Cr-Os t_{2g} basis (in red) in comparison with full non spin-polarized DFT band structure (in green). The zero of the energy is set at Fermi energy. The energies, E0 and E1 represent the energy points about the expansions were carried out in NMTO calculation.

Note this low energy model does not include the majority spin Cr t_{2g} orbitals. Thus their small but finite hybridization with the Os t_{2g} orbitals is neglected. Consequently the bandwidths of the Os t_{2g} spin-split bands and the Cr minority spin t_{2g} bands included in the model calculation are not expected to agree perfectly with DFT. This however is not going to effect the physics of obtaining a half-metal state upon electron (La) and hole (Na) doping as long as the band positions with respect to the Fermi energy are reproduced correctly. Note that the energy scales of the model Hamiltonian, namely Δ = ϵ_{Cr} - ϵ_{Os}, the spin-independent hopping parameters, as well as $J^{'}$, the energy scale determining the superexchange interaction between Cr and Os spins, all have been estimated from the DFT calculations. As a result the charge-transfer gap between the minority spin Os and Cr t_{2g} bands, given by Δ, and the exchange gap between the Os minority and majority bands, given by $J^{'}$, are reproduced rather accurately.The density of states obtained from

the full model Hamiltonian, as given in Eqn(1) including the J and $J^{'}$ terms is shown in Fig.S2 in comparison to spin-polarized DFT density of states. Inclusion of spin-polarization makes the compound insulating with the gap in down spin channel given by $|\Delta|$ and the gap in up spin channel being driven by spin-splitting within Os t2g states, given by $J^{'}$. The position of the Fermi energy appears correctly in the model calculation situated in the spin gap between the minority and majority Os t_{2g} bands, as found in spin-polarized DFT density of states.

Since the band positions with respect to the Fermi energy are reproduced correctly, the stabilization of a half-metal state upon electron (La) and hole (Na) doping, as found in DFT, is captured accurately in the model calculation. The change of sign of the polarization, obtained using the model Hamiltonian, from the electron doped to the hole doped side, as seen in unbiased DFT calculation, also emerges correctly as the Fermi energy upon doping resides in the majority spin Os t_{2g} band in one case, and in the minority spin Os t_{2g} band in the other case.

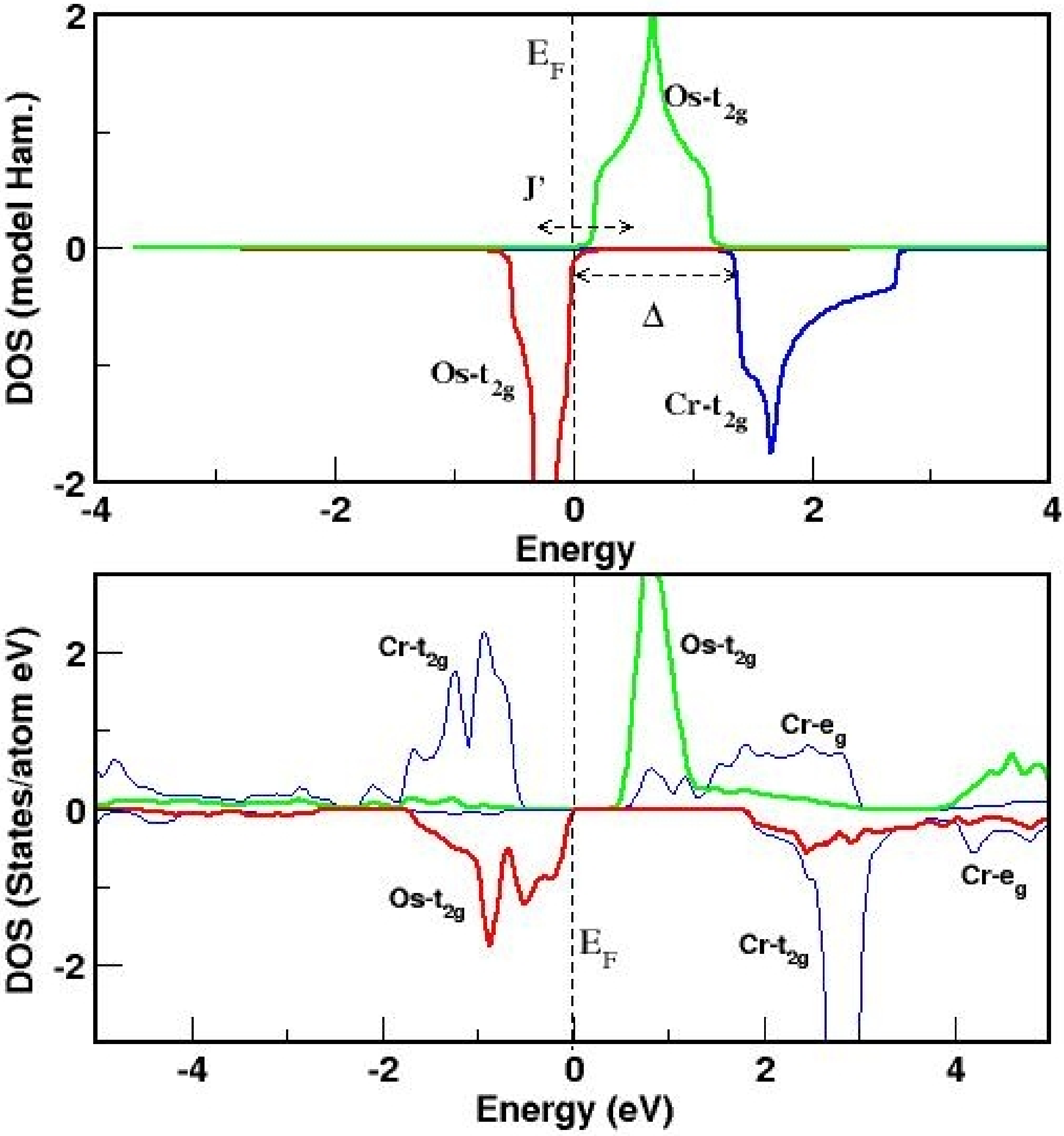

FIG. 2: The density of states obtained in model Hamiltonian calculation for the undoped compound (top panel) and the spin-polarized DFT density of states (bottom panel). Note the majority spin Cr t_{2g} states are missing in model density of states, thus influencing the bandwidths, but the band positions as well as the position of Fermi level are reproduced correctly.
